# Treatment of Typhoid Fever

**Published:** 1902-07

**Authors:** C. T. Nolan

**Affiliations:** Marietta, Ga.


ATLANTA

### Journal-Record of Medicine.

Successor to Atlanta Medical and Surgical Journal, Established 1855, and Southern Medical Record, Established 1870.

VOL. IV. JULY, 1902. No. 4.

BERNARD WOLFF, M.D., EDITOR, Nos. 319–20 Prudential.

Published Monthly.

M. B. HUTCHINS, M.D., BUSINESS MANAGER, No. 64 Marietta St.

### *ORIGINAL COMMUNICATIONS.*

### TREATMENT OF TYPHOID FEVER.*

BY C. T. NOLAN, M.D.,
MARIETTA, GA.

Owing to the prevalence of typhoid fever its treatment is always a subject of importance and interest to the medical profession. In one of our recent works on the practice of medicine, an eminent author says: "In hospital practice medicines are not often needed. A great majority of my cases do not receive a dose." This is, I think, an extreme view to take of the treatment of typhoid fever. Under this do-nothing plan a trained nurse could treat the case and the services of the physician not be needed at all. On the other hand, we have those who give entirely too much medicine in the treatment of their cases. I believe that medicine given judiciously will not only lessen the severity of the disease, but will, in the majority of cases, cut short its duration five or six days.

*Read before the Medical Association of Georgia, Savannah, April, 1902.

For the last five years I have been treating typhoid fever with the combined antiseptic and eliminative plan. During this time I have treated thirty-eight cases, and out of this number four had intestinal hemorrhages, two relapsed, and two died. The two deaths occurred during my treatment of fourteen cases the past year. I regret that very accurate notes of these cases were not kept, but I am sure that in four of these the temperature was normal by the fourteenth day; in eight, by the sixteenth; in twenty, by the eighteenth; and in two the temperature lasted more than twenty-eight days.

Prior to my present mode of treatment of typhoid fever, a majority of my patients did not have a normal temperature before the twenty-second or the twenty-eighth day. When I first began the Woodbridge treatment I followed his instructions very closely, except I gave larger doses and at longer intervals. I found that under this treatment the purging was too severe, and consequently I have eliminated what I consider the objectionable features of that treatment.

One will rarely meet with a disease in the treatment of which a greater draft will be made upon the physician's skill and sound judgment than that of typhoid fever. And in the treatment of this disease the symptoms must be met as they unfold themselves, but one should prevent their occurrence and not stand idly by and wait for some bad or fatal symptom to present itself before making an effort to give the patient the benefit of medicine.

I will now give in a general way my plan of treatment: At first I give four grains each of calomel and bicarbonate of soda in four doses one hour apart. Then I begin with either salol or carbonate of guaiacol, given in five-grain doses every four hours, which is continued throughout the entire treatment of the disease, unless there is some special reason for discontinuing it. The only bad effect that I have ever had from the administration of salol was in the case of a negro boy ten years old, who was taking three grains every four hours for a period of ten days. The result—his urine was changed to a very dark color.

As a stomachic and routine treatment, I gave ten drops of tincure of nucis vomicæ three times a day. The calomel and soda, one grain each, is given in two doses one hour apart every other day throughout the treatment of the disease, unless the diarrhea reaches the stage where it forbids its use. I do not believe the calomel is contraindicated unless there is more than five evacuations of the bowels within twenty-four hours. In fact I should prefer that the bowels should act only three or four times every twenty-four hours. But should the diarrhea reach the stage where it weakens the patient, say six or seven movements within twenty-four hours, then the calomel should be discontinued and large doses of bismuth given. I never give opium for the diarrhea if it can well be avoided, because it locks up the secretions, and I believe it is the *sine qua non* in the treatment of typhoid fever to keep the secretions thoroughly open. In a few instances it has been found necessary to give hypodermics of morphia in cases where there was extreme restlessness and nervousness after the cold bath and sulphonal had failed to produce quietness and sleep. The nurse is always instructed to give the patient large quantities of water to drink. I make it a rule to examine the feces often to see if the food is being well digested; if not, I put lime-water with the milk or change to buttermilk. Ice-cream can often be given with much benefit to the patient. After the tenth day I almost invariably give liquid peptonoids. While the nourishment of the patient should be looked after most carefully, I do not believe in giving solid food as some writers of to-day advocate; but the diet should be liquid, though varied as much as possible and given at stated intervals.

As to the reduction of the temperature, I rely principally on the cold sponge bath. Occasionally I give some antipyretic, preferably ammonol and acetanilid. We often hear physicians say that they cannot use water for the reduction of temperature, because they affirm it can only be done by trained nurses in our sanitariums. The bath can be given in private practice and with great benefit to the patient if the doctor will give one or two sponge baths in order to instruct those in charge of the patient, should they not know the manner in which it is given. You place a rubber sheet under the patient, and make an improvised tub by putting quilts on either side of the patient and under the sheet. For the first five minutes use warm water with sponges and active massage, except of course over the abdomen, then gradually add ice until the temperature of the water is about seventy Fahrenheit. Continue the sponging and massage at least fifteen or twenty minutes more, then remove the sheet, cover the patient well, and if necssary, put hot-water bag to his feet. As a rule your patient will fall asleep and wake much refreshed.

The coal-tar derivatives, like all drugs that are positive in their action, can do much harm as well as good, and I believe the pendulum has swung too far in the condemnation of these remedies. If the temperature persists in remaining high, say 103.5 to 105 in spite of the baths; or, if the baths have to be given so frequently as to be too fatiguing to the patient, then it is that, if the condition of the heart will at all allow it, the antipyretics must be given. Five grains of ammonol will usually reduce the temperature two degrees or more, and the patient will, for the time being, be much benefited. Ammonol does not seem to depress the heart's action as much as the other antipyretics. I would not give ammonol or any other antipyretic if the pulse was weak and dicrotic; but given cautiously they are a valuable aid and almost indispensable in the treatment of typhoid fever.

As to stimulants, I rely upon strychnin and whiskey, but do not give either unless there is positive indication for their use. I have made patients very nervous by overstimulation with strychnin and was forced to discontinue its use.

I will briefly report my thirty-eighth case: On the 27th of October last I was called to Mrs. G. E. L., wife of a physician, age twenty-eight years. She had been ill for two days but had not taken her bed, suffering with severe headache, loss of appetite, heavily coated tongue, tenderness over abdomen, temperature 103, pulse 100. I believed at this time my patient had typhoid fever, and later developments confirmed this belief. On the sixth day of her illness the characteristic rash of typhoid fever literally covered her body. At this time her temperature was 105, pulse 120, marked tympanitis, tongue brown and dry, great accumulations of sordes on her teeth. She was unable to empty her bladder and the catheter had to be used from the fourth to the fourteenth day.

In the main the treatment as outlined above was given in this case, and her temperature was normal by the fifteenth day. This case was virulent, and I believe, under the ordinary expectant plan, would have lasted three and one half weeks.

In conclusion, I wish to say I am fully aware that the eliminative treatment of typhoid fever does not meet the approbation of a large majority of the profession; but I believe the eliminative treatment, whereby we assist nature, the toxic products of typhoid fever being eliminated through the alimentary canal and other emunctories of the body, is a rational one, and that its advocates will increase in the future treatment of typhoid fever.

